# Neuroprotective Effect of Resveratrol Against Methamphetamine-Induced Dopaminergic Apoptotic Cell Death in a Cell Culture Model of Neurotoxicity 

**DOI:** 10.2174/157015911795017353

**Published:** 2011-03

**Authors:** Kavin Kanthasamy, Richard Gordon, Huajun Jin, Vellareddy Anantharam, Syed Ali, Anumantha G Kanthasamy, Arthi Kanthasamy

**Affiliations:** 1 Dept. of Biomedical Sciences, Iowa Center for Advanced Neurotoxicology, Iowa State University, Ames, IA 50011-1250; 2 Neurochem. Lab., Div. of Neurotox., NCTR/FDA, Jefferson, AR, USA

**Keywords:** Resveratrol, drug abuse, neuroprotection, neurotoxicity, oxidative stress, apoptosis.

## Abstract

A growing body of evidence suggests that oxidative stress-mediated cell death signaling mechanisms may exert neurotoxic effects of methamphetamine (MA)-induced dopaminergic neuronal loss. However, the means by which oxidative stress induced by MA causes neurodegeneration remains unclear. In recent years, resveratrol has garnered considerable attention owing to its antioxidant, anti-inflammatory, anti-aging, and neuroprotective properties. In the present study, we sought to investigate the neuroprotective effects of resveratrol against apoptotic cell death in a mesencephalic dopaminergic neuronal cell culture model of MA neurotoxicity. MA treatment in the N27 dopaminergic neuronal cell model produced a time-dependent activation of the apoptotic cascade involving caspase-3 and DNA fragmentation. We found that the caspase-3 activation preceded DNA fragmentation. Notably, treatment with resveratrol almost completely attenuated MA-induced caspase-3 activity, but only partially reduced apoptotic cell death. We conclude that the neuroprotective effect of resveratrol is at least in part mediated by suppression of caspase-3 dependent cell death pathways. Collectively, our results demonstrate that resveratrol can attenuate MA-induced apoptotic cell death and suggest that resveratrol or its analogs may have therapeutic benefits in mitigating MA-induced dopaminergic neurodegeneration.

## INTRODUCTION

Methamphetamine (MA) is a psychostimulant that is widely abused in the US and worldwide [1]. Repeated use has been shown to be associated with euphoria, decreased appetite, increased alertness, and hyperthermia [2]. Several of these effects are believed to play a central role in the high abuse potential of MA, which causes not only severe health problems but also poor socioeconomic conditions for the abusers. MA use leads to functional alterations and neurodegenerative changes in various brain regions [3, 4]. Notably, decreased levels of tyrosine hydroxylase (TH) and dopamine transporter (DAT) immunoreactivity were measured in the striatum of MA users [5, 6]. Additionally, several studies have repeatedly demonstrated that in rodents MA causes long term neurodegenerative changes in dopaminergic and serotonergic nerve terminals in several brain regions, including the cortex, striatum, and hippocampus [7-9]. The changes include long term reductions in striatal dopamine (DA) and serotonin (5-HT) levels, decreased tryptophan hydroxylase and tyrosine hydroxylase, loss of DA and 5-HT transporters, and associated neurodegenerative changes in the monoaminergic terminals [10]. The loss of DA transporters has been documented in abstinent MA users; this deficiency is believed to contribute to reduced motor function and memory impairments [11]. Furthermore, based on the similaritiesbetween MA-induced dopaminergic neurodegenerative changes and PD-induced neuronal damage, MA has been used as a putative model for the study of dopaminergic terminal degeneration associated with Parkinsonism [12]. 

Although several mechanisms have been proposed to underlie MA-induced neurotoxicity, the exact mechanism remains unknown. Mounting evidence suggests that elevated oxidative stress may contribute to MA-induced degenerative changes. In fact, oxidative damage to proteins, lipids and DNA has been shown in the brains of MA-treated animals [13-15]. Accumulation of oxidative products may lead to activation of the apoptosis related cell death cascade. Increasing lines of evidence suggest that mitochondria-dependent apoptotic signaling events may underlie neurodegenerative changes associated with MA-induced neurotoxicity [16, 17]. Experimental evidence indicates that key events of MA-induced mitochondrial changes include membrane depolarization, Bcl2/Bax dysregulation, cytochrome C release, and capase-9 and -3 activation [18, 19]. Moreover, administration of antioxidants effectively attenuated key MA-induced mitochondrial dependent apoptotic changes, suggesting that oxidative stress plays a role in the neurotoxicity [20-22].

Resveratrol (3,5,4’-trihydroxystilbene) is a polyphenolic phytoalexin found in the skin of red grapes, red wine, and other natural food sources such as nuts. Interestingly, resveratrol has been shown to possess a wide variety of biological and pharmacological effects, including antioxidant, anti-apoptotic, anti-inflammatory, anti-carcinogenic, and anti-aging properties [23-27] Furthermore, resveratrol has been shown to attenuate neuronal death associated with MPP^+^-induced oxidative stress [28-31]. However, currently there is no information available regarding the neuroprotective effects of resveratrol on MA-induced dopaminergic neurodegenerative effects. Hence, we hypothesized that resveratrol may also protect dopaminergic neurons against MA-induced neurotoxicity by modulating the caspase-3 dependent apoptotic cell death pathway. Therefore, we evaluated possible neuroprotective properties of resveratrol in a well characterized cell culture model of MA -induced dopaminergic neurotoxicity. 

## MATERIALS AND METHODS

### Materials

Methamphetamine was obtained from the National Institute of Drug Abuse (NIDA) drug supply program. Caspase-3 substrate, Ac-DEVD-AFC, was purchased from EMD Biosciences, Inc. (Gibbs town, NJ). Cell Death Detection Elisa Plus Assay Kit was purchased from Roche Molecular Biochemicals (Indianapolis, IN). All tissue culture supplies were purchased from Gibco-BRL (Gaithersburg, MD). Other routine laboratory reagents were purchased from Fisher Scientific (Pittsburg, PA). The immortalized rat mesencephalic (N27) cell line was a kind gift of Dr. Kedar N. Prasad, Univ. of Colorado Health Sciences Center (Denver, CO).

### Cell Culture Model

Immortalized rat mesencephalic neuronal cells (1RB_3_AN_27_, commonly referred to as N27 dopaminergic cells) were grown in RPMI medium supplemented with 10% fetal bovine serum, 1% L-glutamine, penicillin (100 U/ml), and streptomycin (100 U/ml), and maintained at 37°C in a humidified atmosphere of 5% CO_2_ (Anantharam, 2002; Clarkson, 1999). We previously reported that N27 neuronal cells can be used as a model system to study the apoptotic changes associated with neurotoxicity of dopaminergic neurotoxicants such as methamphetamine and MPP+ [32-34]. After 2-4 days in culture, N27 cells were harvested and resuspended in complete growth medium at a cell density of 1-3 x 10^6^/ml. Cells were treated with 2 mM MA in the presence or absence of 10 µM resveratrol for 12 or 24 hr. Following the treatments, cells were homogenized and the lysates were used for caspase-3 and DNA fragmentation assays.

### Measurement of Caspase-3 Activity

Caspase-3 activity was used as an early marker of apoptosis and was determined as previously described in our publications [35]. Briefly, cell lysates were then incubated at 37°C for 20-30 min to allow complete lysis. Lysates were quickly centrifuged and cell-free supernatants were incubated with 50 µM Ac-DEVD-AFC (caspase-3 substrate) at 37°C for 1 hr. Caspase activity was then measured by determining the formation of 7-amido-4-trifluoromethylcoumarin (AFC) resulting from caspase cleavage using a Biotek plate reader (excitation 400 nm, emission 505 nm). The fluorescence signals from the samples were normalized to protein concentration, which was measured by the Bradford protein assay.

### DNA Fragmentation Analysis

DNA fragmentation assay was performed using a Cell Death Detection Elisa Plus Assay Kit, as described in our publications [35, 36]. Briefly, 20 µl aliquots of supernatant obtained from cell lysates were dispensed to streptavidin-coated 96 well microtiter plates followed by addition of 80 µl of antibody cocktail. Plates were incubated for 2 hr at RT with mild shaking. The antibody cocktail consisted of a mixture of anti-histone biotin and anti-DNA-HRP directed against various histones and antibodies to both single strand DNA and double strand DNA, which are major constituents of the nucleosomes. After incubation, unbound components were removed by washing with the incubation buffer supplied with the kit. Quantitative determination of the amount of nucleosomes retained by anti-DNA-HRP in the immunocomplex was conducted spectrophotometrically with ABTS as an HRP substrate (supplied with the kit). Measurements were made at 405 nm against an ABTS solution as a blank (reference wavelength ~490 nm) using a Biotek microplate reader.

## RESULTS

### Effects of Resveratrol on Methamphetamine-Induced Caspase-3 Activation 

We previously demonstrated that caspase-3 activation is one of the key events in dopaminergic neurotoxicity caused by several neurotoxicants, including MA and MPP+ [32-34]. We also showed that caspase-3 activity can be used as a reliable marker for evaluation of neuroprotective efficacy of experimental pharmacological agents against diverse neurotoxicants [34-36]. Therefore, we first examined whether resveratrol attenuates MA-induced caspase-3 activation. As depicted in Fig. (**1**), treatment of N27 cells with 2 mM MA resulted in a significant increase (~130% increase; p<0.01) in caspase-3 activation as compared to the control group. However, cells that were pretreated with resveratrol for 1 hr prior to MA treatment showed significantly reduced caspase-3 activation as compared to MA treated cells. The resveratrol alone treated group did not show any significant changes in caspase-3 activation as compared to the control. These results indicate that resveratrol can attenuate activation of key proapoptotic changes following MA treatment. 

### Effects of Resveratrol on Methamphetamine -Induced DNA Fragmentation 

In order to determine whether resveratrol protects against MA-induced neuronal apoptosis, we measured DNA fragmentation in resveratrol treated cells. As shown in Fig. (**2**), MA significantly increased DNA fragmentation following a 24 hr treatment. The magnitude of increase in DNA fragmentation was about 2-fold in MA-treated cells as compared to the control group. We observed a significant reduction in DNA fragmentation in N27 cells pretreated with 10 uM resveratrol for 1 hr prior to MA treatment. In contrast, the resveratrol alone treated group did not have altered basal levels of DNA fragmentation. Collectively, these results demonstrate that resveratrol protects against MA-induced apoptosis in dopaminergic neuronal cells.

## DISCUSSION

In the present study we examined the neuroprotective effects of a polyphenolic natural product, resveratrol, on MA-induced neurotoxicity. We show that pretreatment of mesencephalic dopaminergic cells with resveratrol conferred neuroprotection against MA-induced cell death. Specifically, resveratrol showed anti-apoptotic effects by blocking both caspase-3 activation and DNA fragmentation. Resveratrol treatment alone did not alter basal caspase-3 or DNA fragmentation, suggesting that low micromolar concentrations of the compound are not toxic to dopaminergic neuronal cells. To our knowledge, this is the first study demonstrating the neuroprotective efficacy of resveratrol against MA-induced neurotoxicity.

It is well known that MA causes long term neurodegenerative changes in dopaminergic and serotonergic nerve terminals in several brain regions including cortex, striatum, and hippocampus [37-39]. Neurotoxic mechanisms implicated in MA-induced neurodegneration include, mitochondrial dysfunction, oxidative stress, ER stress, and apoptosis. In fact, the critical contribution of oxidative stress related mechanisms in MA-induced dopaminergic neuronal degeneration has been described extensively, whereby accumulation of oxidatively damaged lipids [40], proteins [14], and DNA [15] has been shown in various brain regions of rodents as well as induction of oxidative stress mechanisms in *in vitro* dopaminergic cell culture models [10]. In addition, MA-induced displacement of DA from vesicles and subsequent buildup within the cytosolic and extracellular space and the resulting formation DA related oxidative product, quinone has been shown to be a critically involved in MA-induced dopaminergic neurotoxicity [41, 42]. Moreover, inhibitors of dopamine synthesis or release can attenuate cellular toxicity in experimental models [43]. Previous studies have suggested that oxidative stress may be an early event in dopaminergic neurodegeneration since neurotoxicity is attenuated by antioxidants such as trolox [44, 45] and glutathione (GSH) [43]. In a recent report, resveratrol inhibited ROS accumulation, depletion of GSH, and cellular oxidative damage following treatment with MPP+ as well as 6-OHDA, suggesting antioxidant factors are important neuroprotective effects of resveratrol [46-48]. Therefore, the contribution of antioxidant properties of resveratrol in preventing MA-induced cell death cannot be discounted.

MA-induced oxidative stress is functionally linked to mitochondrial dependent apoptosis, which has been proposed to play a central role in mediating neurotoxicity [18, 38]. MA is a cationic lipophilic molecule that diffuses into mitochondria and is retained there, resulting in dissipation of the mitochondrial membrane potential and disturbance of mitochondrial biogenesis [17]. Additionally, MA causes increases in pro-apoptotic proteins, namely Bax, Bad, and Bid, and decreases in anti-apoptotic proteins, Bcl-2 and Bcl-XL [18, 19]. Subsequently, release of mitochondrial cytochrome C, followed by activation of caspase-9 and -3, and breakdown of several proteins, including PARP, lamin, and DNA fragmentation factor 45 fragment (DFF-45) [18, 38] have been shown to participate in MA-induced apoptotic cell death. In this context, over-expression of Bcl-2 and inhibition of caspases confers resistance against MA-induced apoptotic cell death [10]. In the mitochondrial dependent apoptotic cascade, caspase-3 activation plays a central role in mediating DNA fragmentation, which ultimately leads to cell death [10, 18, 32, 38]. These studies underscore the importance of the mitochondrial mediated caspase cascade in MA-induced neurotoxicity. In the present study, resveratrol pretreatment almost completely inhibited MA-induced caspase-3 activation, but only partially inhibited MA-induced DNA fragmentation. These results suggest that MA-induced neurotoxicity is not entirely dependent on caspase-3 activation, and that other factors might play a role in the neurotoxicity. ER stress, ubiquitin dysfunction, and autophagic impairment may contribute to cell death. We recently reported that MA dramatically increases autophagy in a dopaminergic cell model [32], but the role of autophagy in MA-induced dopaminergic neuronal loss is currently being studied in our laboratory.

Recent studies have demonstrated that polyphenolic compounds exert protective effects against DA associated oxidative damage in dopaminergic neurons both in *in vivo* and *in vitro* models of dopaminergic neuronal degeneration [48-50]. Resveratrol has multiple pharmacological properties: antioxidant, anti-inflammatory, cardioprotective and anti-aging properties [51]. Emerging studies indicate that resveratrol extends the lifespan *via* the sirtuin pathway [52]. Resveratrol’s antioxidant properties might be partly mediated by increases in SOD and catalase activity [52]. Nevertheless, the antioxidant property alone cannot account for its neuroprotective effects. Resveratrol may activate cell specific signaling pathways that facilitate the activation of prosurvival mechanisms and enable the maintenance of mitochondrial integrity, thereby attenuating the multiple cell signaling pathways that cause cell death. Since multiple complex mechanisms mediate neurodegenerative processes, pharmacological agents like resveratrol, with broad spectrum biochemical properties, may hold promise for use as neuroprotective therapies. Pharmacological agents that target a specific mechanism often fail or are ineffective in clinical trials, possibly because of their narrow range of activity.

In summary, we demonstrate that resveratrol treatment is effective against the MA-induced apoptotic cell death process involving activation of caspase-3 and DNA fragmentation. Elucidation of the underlying mechanism of resveratrol neuroprotection in MA-induced apoptotic cell death may improve understanding of the molecular basis of this polyphenolic compound. Evaluation of the molecular mechanisms mediating the neuroprotective effects of resveratrol and other related polyphenols may lead to the development of novel therapies for the treatment of dopaminergic neurodegenerative processes associated with drugs of abuse as well as chronic neurodegenerative disorders including Parkinson’s disease.

## Figures and Tables

**Fig. (1). Resveratrol protects against methamphetamine-induced caspase-3 activity in a dopaminergic neuronal cell model.**
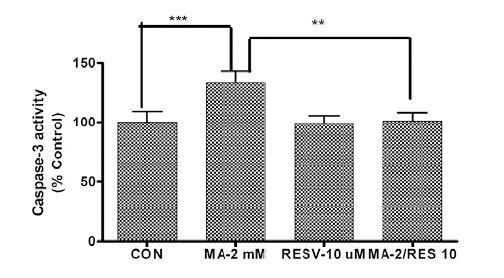
N27 dopaminergic neuronal cells were pretreated with 10 µM resveratrol for 1 hr and then exposed to 2 mM methamphetamine (MA) for 12 hr. Caspase-3 activity was measured using the caspase-3 specific substrate Ac-DEVD-AFC, as described in “Materials and Methods.” Each bar represents mean ± SEM from four individual measurements. *p<0.01, comparison of control vs. MA treatment. **p<0.01, comparison of MA vs. MA and resveratrol treatment.

**Fig. (2). Resveratrol attenuates methamphetamine-induced DNA fragmentation in a dopaminergic neuronal cell model.**
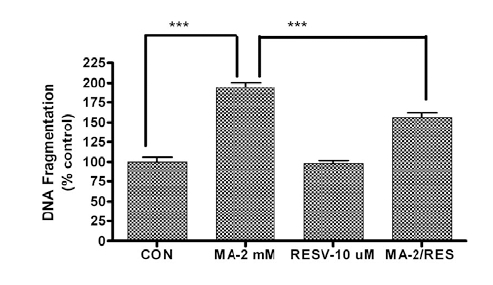
N27 dopaminergic neuronal cells were pretreated with 10 µM resveratrol for 1 hr and then exposed to 2 mM methamphetamine (MA) for 24 hr. DNA fragmentation was measured using the ELISA based cell death assay kit, as described in “Materials and Methods.” Each bar represents mean ± SEM from four individual measurements. *p<0.01, comparison of control vs. MA treatment. **p<0.01, comparison of MA vs. MA and resveratrol treatment.
